# An Improved Approach for the Control Measurements of a Ski-Flying Hill Inrun: A Case Study of Planica

**DOI:** 10.3390/s20092680

**Published:** 2020-05-08

**Authors:** Tilen Urbančič, Oskar Sterle, Klemen Kregar

**Affiliations:** Faculty of Civil and Geodetic Engineering, University of Ljubljana, Jamova cesta 2, 1000 Ljubljana, Slovenia; oskar.sterle@fgg.uni-lj.si (O.S.); klemen.kregar@fgg.uni-lj.si (K.K.)

**Keywords:** control measurements, crane rails, flying hill inrun, Planica

## Abstract

Ski jumping hills should be prepared for competitions in accordance with project documentation in order to ensure safe and fair conditions for competitors. Geodesy/surveying is essential for guiding preparations and controlling the actual shape of the hill. In this article, we present a methodology for the control measurements and preparation of an inrun for a ski-flying hill in Planica. On each side of the track, there is metal tube that guides the trolley, which mills tracks into the ice. A special platform containing three measuring prisms was designed to control the position of the tubes. The proposed method was thoroughly analyzed in terms of its measurement quality and compared to previously used methodologies. The empirical results suggest that our proposed platform provides inrun geometry with a higher quality than previously used methods.

## 1. Introduction

The international ski federation (FIS) specifies the technical requirements for the design of ski jumping hills very precisely. These requirements are meant to ensure fair and safe competitions on these large sports objects. In this article, we investigate a method to measure the shape of the inrun part of the hill, either as a support for the preparation of the hill before competitions or for control measurements for FIS certification or approval.

The geodetic method for the determination of flying hill inrun geometry is based on the classical surveying polar method, where precise surveying equipment is used to measure the polar coordinates of significant points on the inrun. Typically, the instrument (total positioning system (TPS)) is set up in the vicinity of object that is to be measured. Significant points of this object are signalized through the measurement prism, and the polar coordinates (namely the horizontal direction, vertical angle, and slope distance) are measured. Usually, some reference points with known or specific positions are also measured to define the coordinate system relative to the object observed.

**Figure 1:**
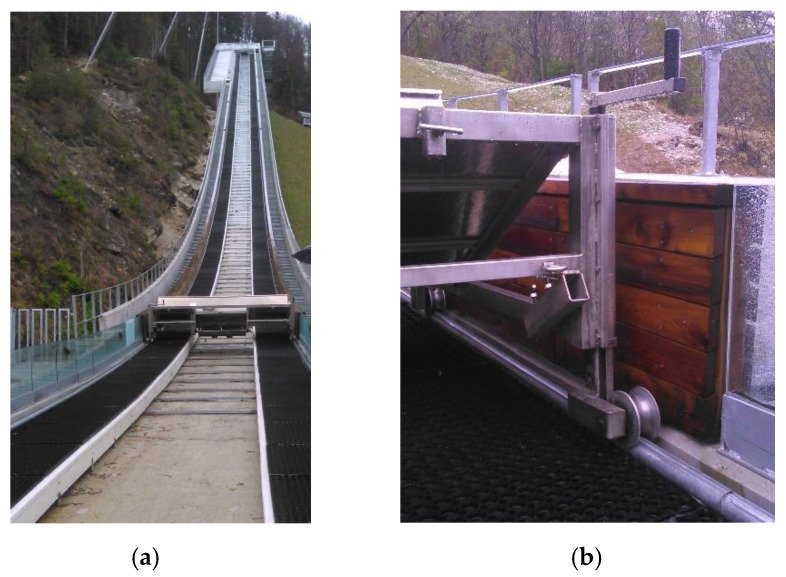
The device for making the snow profile (the trolley): (**a**) inrun of the Planica ski-flying hill; (**b**) installation of the trolley on the tube.

**Figure 2:**
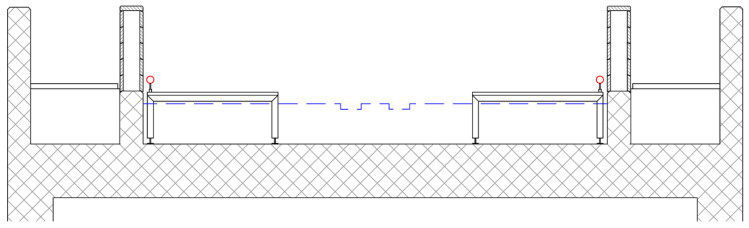
Cross section of the inrun; the guiding tubes are colored red.

The Gorišek brothers’ flying hill in Planica, Slovenia, is one of six ski-flying hills in the world. It usually hosts the last competition of the FIS Ski Jumping World Cup each year. In this article, we study the inrun part of the ski-flying hill. FIS rules [1] determine the shape, length, and gradients of the inrun section. The inrun consists of a straight line at the beginning, two transition curves in the middle, and a straight line at takeoff (the end). The designed geometry is practically implemented through two metal tubes with diameters of 50 mm that run along each side of the track. During preparation of the hill for the competition, the trolley (Figure 1) is driven along the tubes and mills tracks into the ice using a special milling device. To ensure consistency of ski-flying hill geometry with respect to its certificate [2], the precision of each determined point coordinate on the inrun tube should be at least on the level of 5 mm (see Figure 2 for a projected cross section of the inrun).

The longitudinal shape of the inrun is, therefore, determined through the tubes. Each tube is fixed to the base with two adjustable screws on each meter. These screws allow calibration of the tubes (vertical and horizontal adjustment) before each competition.

### 1.1. Overview

**Figure 3:**
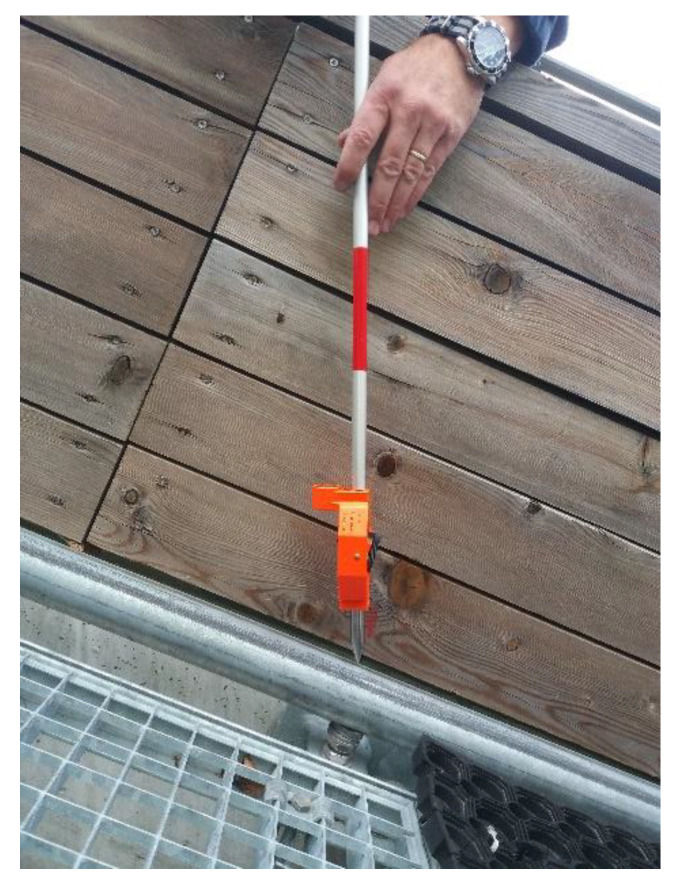
Measurement with the Leica GMP111-0 mini prism.

The geometry of the inrun is currently measured with the classical surveying polar method. The uppermost point on the tube is signalized through a single measurement to a Leica GMP111-0 mini prism (Leica Geosystems, Heerbrugg, Switzerland) (Figure 3) on each meter of the tube. The most problematic is positioning of the rod on the uppermost point of the tube, since it is in no means stabilized or clearly identifiable point, so its identification depends on the surveyor’s subjective judgement. Additionally, Figure 3 also shows how the inclination of the tube imposes a problem of determining the center point/line of the tube. The center point is accessible only with the known inclination angle.

Flying hills are unique objects. Due to their peculiarities, we found no scientific publications that study their control measurements. However, this problem is comparable to the control measurements of the crane rails, with two differences: The rail profiles are different, and the rails do not run horizontally.

The positions of the crane rails are classically controlled with geometric leveling to ensure vertical consistency or height differences between the rails and geometric alignment to ensure parallelism or a gap between the rails [3]. Alternative methods for control measurements have also been reported. Rails can be measured from a geodetic network established on the ground, but a specific signalization of the points on the rail is needed [4]. The same is possible using a single TPS station with improved signalization [5]. Inductive transducers are attached to the trolley, where the position of the trolley is controlled with TPS. This method detects deformations of the rail in great detail [6,7]. A laser can be used to provide a reference line, and a receiving sensor, carried by the trolley, detects the unevenness of the rail [8]. The most complex method for controlling crane rails is using sensorized trolleys or monorails equipped with additional sensors (cameras, lidar, inclination sensors, laser trackers, etc.) [9,10,11]. All proposed methods assume the horizontal position of the rails and are, therefore, not suitable for controlling the geometry of the flying hill inrun.

Tracks can also be measured with laser scanning technology [12,13,14,15,16], but, due to steep base and lengths of flying hills, the precision and resolution of point clouds is insufficient.

### 1.2. Focus and Outline

In this article, we present a method based on the classic polar survey method. We develop and use an improved multi-prism platform developed specifically for measuring and determining the center of a round tube, referred to as the characteristic point of the tube. We mainly focus on the design of the platform and developing a mathematical model for characteristic point determination and quality assessment, all based on the least squares method. We test the platform and methodology in a laboratory on a 1.4 m straight tube, where the geometry of the tube is known. In the case study section, we use the platform on the inrun of the Planica flying hill. In both cases (laboratory and Planica), the platform results are compared to the results of the currently used polar method.

## 2. Methods

Within this section, we present the proposed methodology for controlling the geometry of the tubes on the flying hill inrun. We start with the design and construction of the new multi-prism platform and its calibration. We continue with the mathematical model definition, where we emphasize on characteristic point determination and its quality assessment. In the following, we compare our methodology to the previously used polar method, denoted as the single-prism method, both analytically and empirically. The computational process for the tube’s center from the observations of the three prisms of the new platform provides comprehensive insight into the quality of the position determination and the shape of the inrun profile.

### 2.1. Proposed Multi-Prism Platform

**Figure 4:**
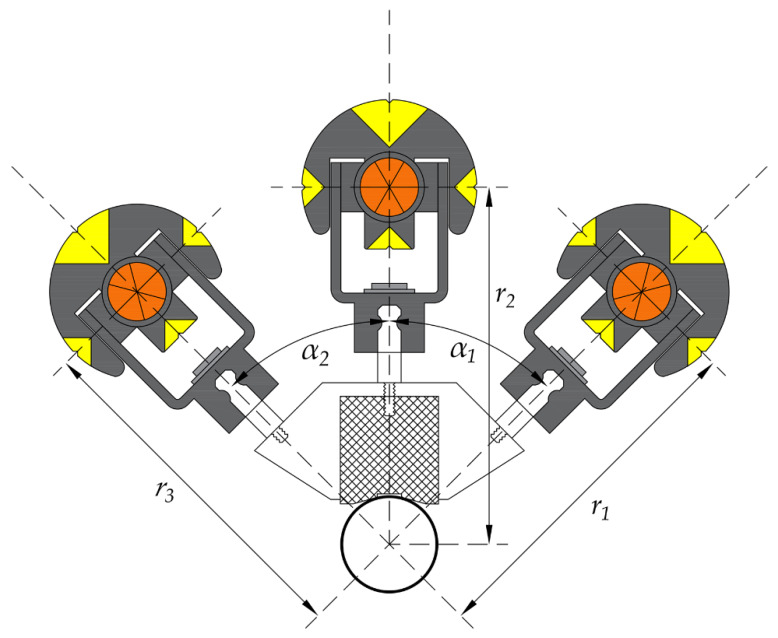
The design of the new multi-prism platform—magnetic base for fixing the platform to the metal tube (hatch fill), the connecting element with prism adapters and the three mini prisms. The geometry of the platform is accurately defined by three distances from the tube center ($r_{1}$, $r_{2}$, and $r_{3}$) and two central angles ($\alpha_{1}$ and $\alpha_{2}$).

This research is based on a newly designed and developed platform, which, together with the developed algorithm, improves the geometry quality of ski-flying hill inrun tubes. This new multi-prism platform consists of a magnetic base for fixing the platform to the metal tube, a connecting element with prism adapters, and three mini prisms (Figure 4). Geometrically, the multi-prism platform should satisfy the condition that the center of the circle through all three prisms coincides with the center of the tube. Unlike the polar method, the multi-prism method always determines the center point/line of the tube.

The platform is attached to the tube by a magnet that is also an integral part of the Bahco micrometer with dimensions of 50 mm × 52.5 mm × 62.5 mm. The bottom surface of the magnet, which was placed on the tube in our experiment, is not flat but instead consists of two inclined planes that sit on the tube. Moreover, this magnet includes M10 thread in the center of its upper surface. The connecting element is made of metal. The dimensions of the connecting element between the magnet and the prism adapters were determined according to the dimensions of the magnet and the used Leica GMP101 mini prisms. In order to ensure quality measurements, it was also important to properly treat the surfaces where the prism adapters are attached. Due to the strict geometrical conditions, the connecting element was manufactured with 1/100 mm precision on a CNC machine.

**Figure 5:**
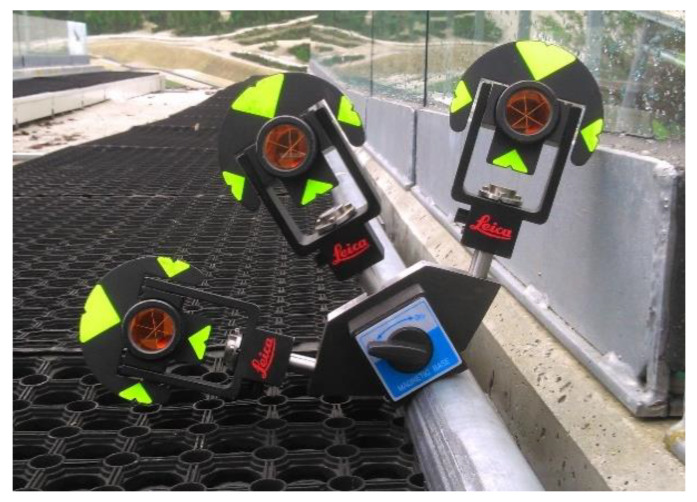
Setup of the new multi-prism platform on a tube.

The prism adapters are 40 mm long with a standard diameter of 12 mm and are made of stainless steel. Their upper sections are designed to allow unique prism placement. For signalization, Leica GMP101 mini prisms were selected. These prisms provide a sufficient quality of measurement, as their declared centering error is only 1.0 mm [17]. The use of more precise round Leica GPH1P prisms was not possible on the platform at the Planica ski-flying hill because of their excessive size (see Figure 2 and Figure 5).

The guiding tube has a diameter of 50 mm. The center of the circle, determined by all three prisms, coincides with the center of the tube. In order to use the proposed multi-prism platform, we calibrated the platform (Section 2.2) and developed a mathematical model to determine the characteristic point of the platform with its precision on the basis of the least squares method (Section 2.3).

### 2.2. Calibration of the Multi-Prism Platform

The true dimensions and geometrical properties of the platform differ slightly from the designed ones and, therefore, need to be determined precisely. The parameters are the distances from the center of the tube to the three prisms ($r_{1},r_{2},r_{3}$) and the two central angles $\alpha_{1}$ and $\alpha_{2}$ (see Figure 4). The geometry of the platform was measured in a laboratory environment with a DEA Gamma 1203 Coordinate Measuring Machine (CMM TECHNOLOGY, San Clemente, CA, USA) [18], where all parameters were determined with accuracy at a 1/100 mm level:

- Radii: $r_{1}=183.366$ mm, $r_{2}=182.988$ mm and $r_{3}=$ 183.770 mm;
- Angles: $\alpha_{1}=44^{{}^{\circ}}24^{\prime }23^{\prime \prime }$ and $\alpha_{2}=45^{{}^{\circ}}24^{\prime }4^{\prime \prime }$.

The calibrated parameters of the platform are used in the following algorithm for the calculation of the tube center based on the measured prism centers. 

### 2.3. Characteristic Point Determination

**Figure 6:**
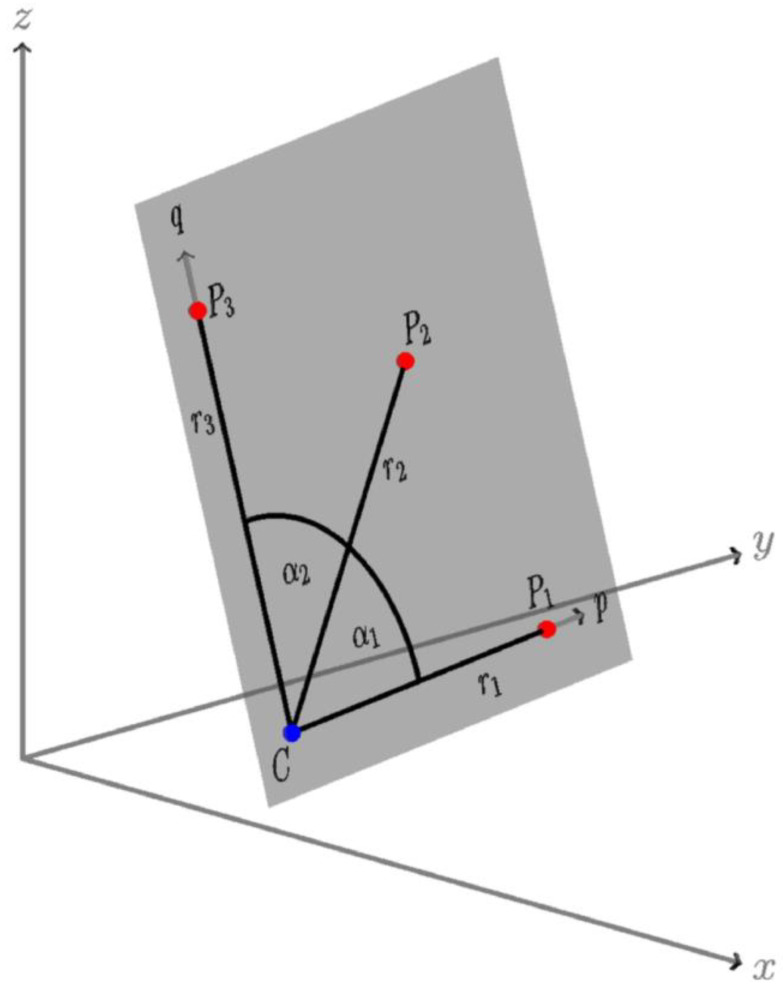
Mathematical model representing the geometry of the new multi-prism platform.

The adjustment of the platform parameters was determined by a mathematical model that relates observations with parameters [19]. This mathematical model is depicted in Figure 6. In our case, the observations represent the coordinates measured with the polar method for all three measurement prisms (points $P_{1}$, $P_{2}$, and $P_{3}$) on the platform and are labeled with $x_{i}={\left[\begin{array}{ccc} x_{i} & y_{i} & z_{i} \end{array}\right]}^{T}$ (i=1, 2, 3). These are object coordinates since they are represented in the object (global) coordinate system. The number of all observations for each setup of the platform is thus n=9. Stochastic information for each object point is represented with the covariance matrix $\Sigma_{i}$. The details on determining the quality of measured object points are represented in Section 2.4. and Section 2.4.1.

The functional model for adjustment is based on rigid-body transformation. Points $P_{1}$, $P_{2}$, and $P_{3}$ are, according to Figure 6, coplanar in the plane of the platform (pq plane), with the accurately known inter-geometry defined by radii $r_{1}$, $r_{2}$, $r_{3}$ and angles $\alpha_{1}$, $\alpha_{2}$. The coordinates of points $P_{1}$, $P_{2}$, and $P_{3}$ in the pq plane are labeled with $p_{i}={\left[\begin{array}{ccc} p_{i} & q_{i} & 0 \end{array}\right]}^{T}$ (i=1,2,3) and are denoted as planar coordinates. These coordinates are defined as

(1) $$p_{1}=\left[\begin{array}{c} r_{1}\\ 0\\ 0 \end{array}\right]\;p_{2}=\left[\begin{array}{c} r_{2}\cos\alpha_{1}\\ r_{2}\sin\alpha_{1}\\ 0 \end{array}\right]\;p_{3}=\left[\begin{array}{c} r_{3}\cos\left(\alpha_{1}+\alpha_{2}\right)\\ r_{3}\sin\left(\alpha_{1}+\alpha_{2}\right)\\ 0 \end{array}\right]$$

Point $C={\left[\begin{array}{ccc} 0 & 0 & 0 \end{array}\right]}^{T}$ in pq plane, in this case, corresponds to the tube center. Since radii $r_{1}$, $r_{2}$, $r_{3}$ determine the scale of the platform, 6 transformation parameters need to be determined to optimally fit the planar coordinates $p_{i}$ onto the measured object coordinates $x_{i}$. The number of parameters in the adjustment is thus u=6, including the translation parameters $C={\left[\begin{array}{ccc} C_{x} & C_{y} & C_{z} \end{array}\right]}^{T}$ and the rotation parameters $\Omega ={\left[\begin{array}{ccc} \omega_{x} & \omega_{y} & \omega_{z} \end{array}\right]}^{T}$ (that define rotation matrix $R=R_{x}R_{y}R_{z}$). The translation parameters C define the coordinates of the characteristic points of the tube on the flying hill inrun, while the rotation parameters define the orientation of the tube at each characteristic point in the object coordinate system. The i-th planar point, $p_{i}$, is related to the i-th measured object point $x_{i}$ as [20]

(2) $$x_{i}=C+Rp_{i}=C+R_{x}R_{y}R_{z}p_{i}.$$

The linearized form of Equation (1), written in the Gauss–Markov model form, is as follows:

(3) $$E\left(\Delta x_{i}\right)=\frac{\partial x_{i}}{\partial C}\delta C+\frac{\partial x_{i}}{\partial \Omega }\delta \Omega \;D\left(\Delta x_{i}\right)=\Sigma_{i}=\sigma_{0}^{2}P_{i}^{-1}$$

where operators E(·) and D(·) stand for the expected value and dispersion, respectively, whereas $\sigma_{0}^{2}$ stands for the reference variance a-priori, and $P_{i}$ is the for full-rank weight matrix for point i. $\Delta x_{i}$ stands for the misclosure (observed-minus-computed) values of the observations, whereas δC and δΩ represent corrections to the approximate values of the parameters [19]. The partial derivatives of all parameters are defined as [20]

(4) $$\begin{array}{l} \frac{\partial x_{i}}{\partial C}=I\\ \frac{\partial x_{i}}{\partial \Omega }=\left[\begin{array}{ccc} \frac{\partial R}{\partial \omega_{x}}p_{i} & \frac{\partial R}{\partial \omega_{y}}p_{i} & \frac{\partial R}{\partial \omega_{z}}p_{i} \end{array}\right] \end{array}$$

The partial derivatives of rotation matrix R for all rotation angles are

(5) $$\frac{\partial R}{\partial \omega_{x}}=\frac{\partial R_{x}}{\partial \omega_{x}}R_{y}R_{z}=L_{x}R_{x}R_{y}R_{z}\;\frac{\partial R}{\partial \omega_{y}}=R_{x}\frac{\partial R_{y}}{\partial \omega_{y}}R_{z}=R_{x}L_{y}R_{y}R_{z}\;\frac{\partial R}{\partial \omega_{z}}=R_{x}R_{y}\frac{\partial R_{z}}{\partial \omega_{z}}=R_{x}R_{y}L_{z}R_{z}$$

Matrices $L_{\iota }$ in Equation (5) represent the linear differential operators for rotation matrices $R_{i}$ (i=x,y,z) and are defined as

(6) $$L_{x}=\left[\begin{array}{ccc} 0 & 0 & 0\\ 0 & 0 & -1\\ 0 & 1 & 0 \end{array}\right]\;;\;\;\;\;\;\;L_{y}=\left[\begin{array}{ccc} 0 & 0 & 1\\ 0 & 0 & 0\\ -1 & 0 & 0 \end{array}\right]\;;\;\;\;\;\;L_{z}=\left[\begin{array}{ccc} 0 & -1 & 0\\ 1 & 0 & 0\\ 0 & 0 & 0 \end{array}\right]$$

The final Gauss–Markov model for all three points ($P_{1}$, $P_{2}$, and $P_{3}$) in each setup of the platform is, therefore, written as

(7) $$E\left(\left[\begin{array}{c} \Delta x_{1}\\ \Delta x_{2}\\ \Delta x_{3} \end{array}\right]\right)=\left[\begin{array}{cc} I & \frac{\partial x_{1}}{\partial \Omega }\\ I & \frac{\partial x_{2}}{\partial \Omega }\\ I & \frac{\partial x_{3}}{\partial \Omega } \end{array}\right]\left[\begin{array}{c} \delta C\\ \delta \Omega \end{array}\right]\;;\;\;\;\;\;\;\;\;\;\;\;\;D\left(\left[\begin{array}{c} \Delta x_{1}\\ \Delta x_{2}\\ \Delta x_{3} \end{array}\right]\right)=\sigma_{0}^{2}\left[\begin{array}{ccc} P_{1}^{-1} & 0 & 0\\ 0 & P_{2}^{-1} & 0\\ 0 & 0 & P_{3}^{-1} \end{array}\right]$$

or, in a compact form, as

(8) $$E\left(\Delta x\right)=A\beta \;;\;\;\;\;\;\;\;\;\;\;D\left(\Delta x\right)=\sigma_{0}^{2}P^{-1}.$$

The design matrix A from Equations (7) and (8) is, according to [20], of full rank because only the transformation parameters are estimated. The solution for parameter vector β and its corresponding cofactor matrix $Q_{\beta \beta }$ is, therefore, straight-forward [19]:

(9) $$Q_{\beta \beta }={\left(A^{T}PA\right)}^{-1}\;;\;\;\;\;\;\;\;\;\;\;\beta =Q_{\beta \beta }A^{T}P\Delta x.$$

The observation residuals v and a-posteriori reference variance ${\hat{\sigma }}_{0}^{2}$ are determined with

(10) $$v=A\beta -\Delta x\;;\;\;\;\;\;\;\;\;\;{\hat{\sigma }}_{0}^{2}=\frac{v^{T}Pv}{n-u}=\frac{v^{T}Pv}{3}$$

For the complete procedure of least squares, a global test is performed [21], and all residuals are checked for outliers [22,23]. Based on the global test result, a decision for reference variance is made to calculate the covariance matrix of parameters $\Sigma_{\beta \beta }$ [19,21].

In order to improve precision of determined characteristic point C, an additional information about the inclination of the tube may be considered in mathematical model. The inclination of the tube $\omega_{I}$ is related to the unknowns in the Gauss-Markov model as:

(11) $$\omega_{I}=\mathrm{asin}\left(\cos\omega_{x}\cos\omega_{y}\right)$$

Including Equation (11) into the Gauss-Markov model will improve estimated precision of adjusted unknowns. Precision of determined inclination angle $\sigma_{\omega_{I}}$ may be determined from designed geometry of the tube as well as from nearby determined characteristic points C. Details on the impact of inclination of the tube on the results of Gauss–Markov model are presented in Section 2.4.2.

### 2.4. Quality Assessment 

We next present a quality assessment for both methods used to determine the characteristic point C. In Section 2.4.1, the quality of the single-prism method (see Section 1.1 and Figure 3) is presented, whereas the quality of the proposed multi-prism platform and methodology is presented in Section 2.4.2.

In both cases, we start by measuring the coordinates of a single prism. Regardless of the method, the position of the prism is measured with the polar method, where the horizontal direction, vertical angle, and slope distance are measured from the total station to the prism. The precision of such measurements depends on the instrument quality defined by ISO 17123 and on the quality of the measuring prism, defined in [17]. 

According to the Deumlich and Staiger classification of surveying instruments [24], only precise instruments are suitable for control measurement tasks. For precise instruments, the standard deviation of the angular measurement is better than 1” and better than1 mm + 1 ppm for the distance measurement. For the maximum distance in our case (130 m) and an instrument of 1” and 1 mm + 1 ppm precision, the longitudinal precision of a single point is 1.13 mm, and the transversal and vertical values are 0.63 mm. For all further quality analysis, we will start with an error sphere of 1 mm for each single point position. 

#### 2.4.1. Quality of the Single-Prism Method

For the single-prism method, depicted in Figure 3, the minimally needed number of measurements is used to determine the characteristic point C (i.e., no redundant observations are obtained, and, therefore, no adjustment can be used). 

We can assess the quality of the characteristic points in this way through error propagation law. The overall precision of a characteristic point is a sum of the following influences:

(i) The quality of the instrument’s position in the chosen coordinate system. When the local coordinate system (defined by instrument itself) is chosen, this effect is eliminated;
(ii) The (im)precision of the distance and angle measurement is a property of the total station instrument defined by ISO 17123;
(iii) Systematic instrumental errors should be eliminated by instrument calibration and the measurement methods;
(iv) Environmental (meteorological) influences are reduced to minimum by applying appropriate correction models for measured meteorological parameters (air temperature, humidity, and pressure) [25,26];
(v) For target point signalization, when the benchmark uniquely defines the position of the target point, the signalization error depends on the (non)verticality of the rod with the measuring prism.

The impact of influence (i) may be eliminated by selecting the local coordinate system. The angle and distance measurement precision of (ii) is briefly presented in Section 2.4. We decided to use a 1 mm error sphere for influence (ii). Using calibrated instruments, proper measurement methods (both faces), and applying appropriate corrections will (mostly) eliminate systematic errors (iii) from and (iv) the observations.

The most important error source lies in target signalization (iv). The nominal centering accuracy [17] of the Leica GMP111-0 prism with a 10 cm prism height is 2 mm. However, more problematic is identifying the uppermost point of the round tube to be measured. We can assume that a skilled surveyor can select the highest point of the 50 mm diameter tube with a precision of 2 mm. Because the positioning of the Leica GMP111-0 prism is done on top of the tube, errors mostly affect the horizontal position, while the vertical component is affected to a lesser extent. 

The overall precision of a uppermost point on a tube measured by the single-prism method ($\sigma_{SP}$) consists of the measurement accuracy of polar method $\sigma_{polar}=1\;\mathrm{mm}$, centering accuracy $\sigma_{cent}=2\;\mathrm{mm}$, and identification accuracy $\sigma_{ident}=2\;\mathrm{mm}$. With the error propagation law, we obtain

(12) $$\sigma_{SP}=\sqrt{\sigma_{polar}^{2}+\sigma_{cent}^{2}+\sigma_{ident}^{2}}=3.0\;\mathrm{mm}$$

The $\sigma_{SP}$ obtained from Equation (12) provides an estimation of quality through which the coordinates the uppermost points of the tube are obtained via the single-prism method. In order to obtain the characteristic point C, radius and inclination of the tube must be considered. In case of no inclination point C is obtained simply by reduction of the height component of the uppermost point on the tube for the radius of the tube. However, in the case of an inclined tube, the simple reduction of the height for the radius will impose a bias in two components, the height and the horizontal component in direction of the tube. Neglecting the inclination will cause a height error with the size of 5 mm and horizontal error with the size of 15 mm for tube radius of 25 mm and inclination of 35° (maximum inclination of Planica ski-flying hill inrun). The inclination must therefore be accounted for in order to achieve precision of polar method of 3.0 mm from Equation (12). Otherwise, only a centimeter level of precision is obtainable with the polar method.

#### 2.4.2. Theoretical Quality of the New Multi-Prism Platform Method

For the quality assessment of the proposed platform, we solve the stochastic part of the Gauss–Markov model described in Section 2.3. We start with covariance matrix $\Sigma_{\beta \beta }$, where we focus on the first half of the matrix that represents the parameters of characteristic point C. However, the numerical values in matrix $\Sigma_{\beta \beta }$ are dependent upon the coordinate system and will change whenever the orientation of the platform changes. To obtain the stochastic properties of C that are unaffected by the selection of the object coordinate system, the error ellipsoid must be determined [19,24]. The latter is obtained via SVD decomposition [27], where three eigenvalues and three eigenvectors are obtained. These eigenvalues are related to the size of the ellipsoid, whereas the eigenvectors are related to the orientation of the ellipsoid.

In order to analyze the quality of characteristic point C, we start with the same quality for all three points $P_{1}$, $P_{2}$, and $P_{3}$ with an error sphere of 1 mm, $\sigma_{PM}=1\;\mathrm{mm}$ (see Section 2.4). We must also consider the error of target point signalization for the Leica prism Mini GMP101, defined by $\sigma_{cent}=1\;\mathrm{mm}$ [17]. On the basis of the error propagation law, the precision of the determined coordinates $\sigma_{MP}$ for each point ($P_{1}$, $P_{2}$, and $P_{3}$) on a multi-prism platform is

(13) $$\sigma_{MP}=\sqrt{\sigma_{polar}^{2}+\sigma_{cent}^{2}}=1.41\;\mathrm{mm}$$

Therefore, the starting points are, according to Equation (13), the measured coordinates for points $P_{1}$, $P_{2}$, and $P_{3}$ and their error spheres with radii of 1.41 mm. Figure 6 depicts this situation. To present the analysis of the stochastic model in a clearer manner, we simulated 5000 measured positions for $P_{1}$, $P_{2}$, and $P_{3}$, which are represented by small red cubes. The object coordinate system was selected according to the following parameters:

- The plane of the platform (pq plane from Figure 6) is parallel to the xz plane (perpendicular to xy and yz plane);
- The direction of the tube is defined with coordinate axis y;
- Point $P_{2}$ is located directly above point C.

Every simulated point is also projected onto all three planes of the object coordinate system (bright-red shadows), where the spherical properties of the stochastic model are clearly seen. The shape of the error ellipse for each point on all three planes is evidently a circle and is represented by a dark-green color. For each simulated triplet ($P_{1}$, $P_{2}$, and $P_{3}$), the methodology from Section 2.3 is applied, and one adjusted position of characteristic point C is determined. All determined positions of point C are depicted with blue cubes, and, again, every position is projected on all three planes (bright-blue shadows). The distribution of all characteristic points is clearly not a sphere but an ellipsoid. The analytical solution to the error ellipsoid of point C is determined from covariance matrix $\Sigma_{\beta \beta }$ [21]. The lengths of all the error ellipsoids’ semi-axes are determined as

(14) $$\sigma_{1}=3.41\cdot \sigma_{MP}=4.8\;\mathrm{mm}\;\sigma_{2}=0.97\cdot \sigma_{MP}=1.4\;\mathrm{mm}\;\sigma_{3}=0.58\cdot \sigma_{MP}=0.8\;\mathrm{mm}$$

The directions for all three lengths are defined as

- The direction of $\sigma_{1}$ (the longest semi-axis) is perpendicular to the xz plane (perpendicular to the pq plane) in the direction of the y axis (along the tube direction);
- The direction of $\sigma_{2}$ (the middle semi-axis) is perpendicular to the yz plane (perpendicular to line $P_{2}$-C);
- The direction of $\sigma_{3}$ (the shortest semi-axis) is perpendicular to the xy plane (in the direction of line $P_{2}$-C).

**Figure 7:**
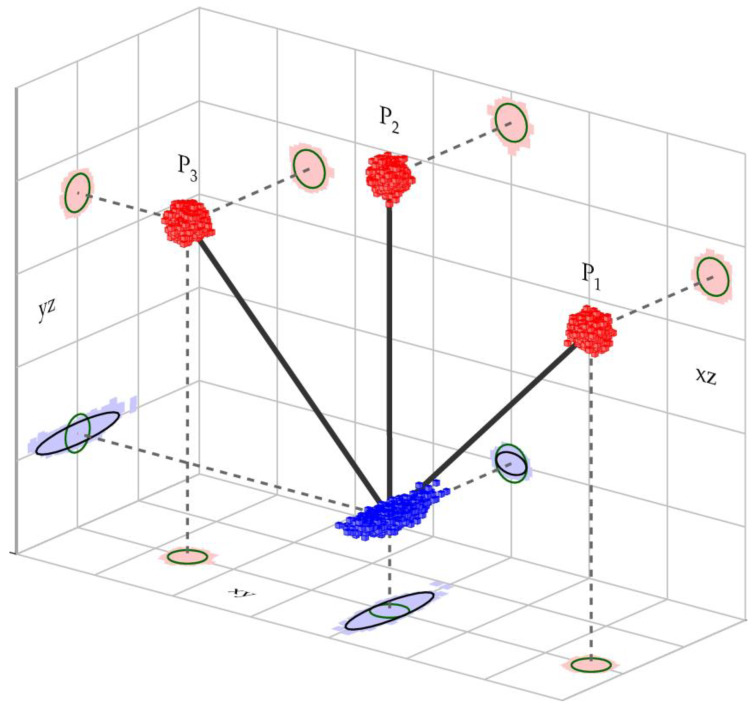
Quality assessment of characteristic point C.

We can see that the proposed multi-prism platform can determine the position of characteristic point C with the following precision Figure 7:

- Along the guiding tube with a precision of $\sigma_{1}=4.8\;\mathrm{mm}$;
- Perpendicular to the guiding tube with
  ○ A precision of $\sigma_{2}=1.4\;\mathrm{mm}$ precision to line $P_{1}$-$P_{2}$;
  ○ A precision of $\sigma_{3}=0.8\;\mathrm{mm}$ parallel to line $P_{2}$-C.

The most important directions for characteristic point C are both the directions perpendicular to the guiding tube direction. In both cases, the precision of the C position is improved with respect to the precision of the measured points, $P_{1}$, $P_{2}$, and $P_{3}$. The improvement increases from 3% to 42%. For the tilted platform set up as depicted in Figure 7, where the platform is tilted by approximately 40° counterclockwise. The improvement of the positional precision in both directions remains around 20% at a 1.1 mm level.

However, we have to consider the inclination of the tube also in the case of multi-prism platform. The inclination of the tube will incline the error ellipsoid from Figure 7 in upward position. This will consequently deteriorate the vertical precision and improve the horizontal (in-line-of-tube) precision. For inclination of 35°, the vertical component will be degraded from 0.8 mm to 2.8 mm, and the horizontal (in-line-of-tube) component will be improved from 4.8 mm to 4.0 mm. The traverse direction is not affected.

In order to reduce the impact of the tube inclination, Equation (11) is included into the Gauss–Markov model. Information about the inclination of the tube will reduce only the largest semi-axis of the error ellipsoid and the level of the reduction is directly related to the precision of the inclination. Table 1 represents the improvement in precision of point C, in the direction of the tube, as a function of inclination precision. The first column represents the precision of the inclination, the second column obtained multiplication factor of nominal precision $\sigma_{MP},$ as expressed in Equation (14), and the last column represents obtained positional precision in the line of the tube.

The first row represents the solution, when no information of inclination is added to the model and the value corresponds to the semi-major axis of the error ellipsoid from Equation (14) and Figure 7. All other rows represent the improvement of the position precision in the line of the tube. Precision of inclination of 2° will for example improve the positional precision from 4.8 mm to 3.7 mm. Results from Table 1 show that when inclination is known with 0.4° precision, then the positional precision is determined on the level of $\sigma_{MP}$. Inclination precision on a level of 0.4° corresponds to a 7 mm change in point position on a distance of 1 m, precision that is possible to achieve also with the multi-prism platform.

Comparing the quality assessments for both methods (the single-prism (Section 3.1) and proposed multi-prism methods), we see that the precision for the determined point C in general increases under a multi-prism approach and decreases with a single-prism method with respect to the precision of the measured object points, mostly due to the redundant observations of the multi-prism platform. Redundant observations enable us to apply a least squares adjustment, where a quality assessment is possible by using residuals, which can also be tested for outliers.

#### 2.4.3. Empirical Quality Evaluation

The empirical quality of the tube center’s determination with the new platform was evaluated by measuring a 1.40 m long straight tube in a laboratory environment. We used a Leica Geosystems TS 30 total station to perform a survey via the polar method from a distance of 3 m. Identical signalization was used for the laboratory test and for the measurements at the case site in Planica.

**Figure 8:**
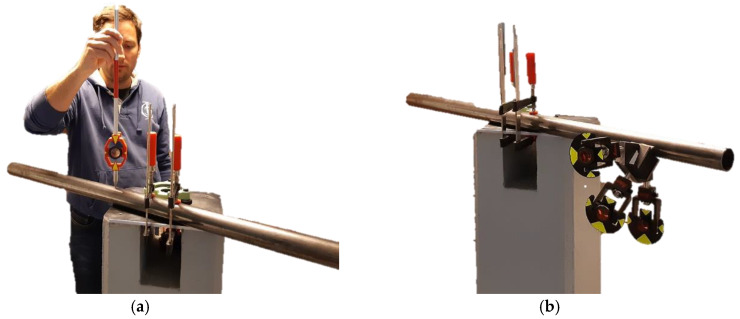
Measurement in a laboratory: (**a**) single-prism; (**b**) new platform.

The test was performed by determining 60 independent positions of the tube center for each method separately (Figure 8):

- Single-prism: positioning of a single-prism 60 times independently at the top of the tube (Figure 8a);
- Multi-prism platform: 60 independent measurements of the platform at different positions along the tube (Figure 8b).

For the single-prism method, the center of the tube was obtained by simply subtracting the prism height and tube radius from the vertical component of the measured point. The tube center coordinates for the multi-prism platform were determined according to the procedure in Section 2.3.

The quality of the tube center determination was evaluated by fitting a line to the determined tube center points for both methods (the single- and multi-prism methods). The final quality evaluation was given by the standard deviations of the residuals and is presented in Section 3.1.

## 3. Results

### 3.1. Empirical Quality Evaluation Results

**Figure 9:**
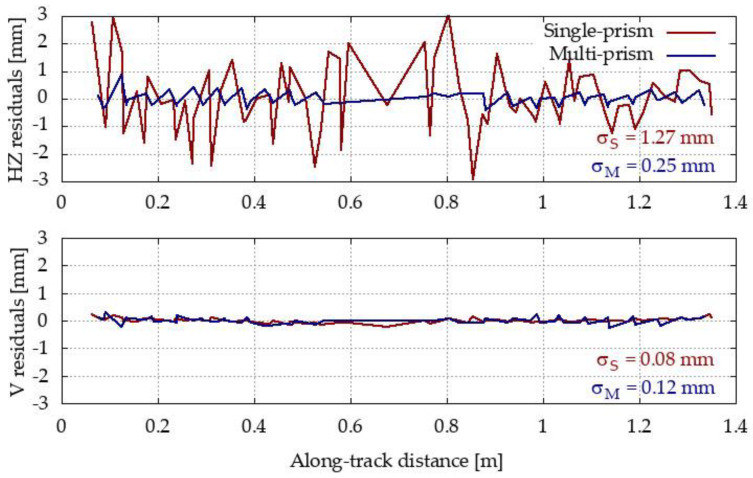
Residuals of the measured values from the adjusted line for the compared methods.

The residuals of the measured centers from the adjusted line are shown in Figure 9. The residuals are shown separately for the vertical and horizontal directions for both methods. The standard deviations were also calculated and are represented in the Figure 9.

For the horizontal direction, the precision of the determined tube centers was significantly better under the multi-prism method (0.25 mm) compared to the single-prism method (1.27 mm). Most of the errors for the single-prism method may be attributed to an error of point identification at the top of the tube. For the vertical direction, the precisions are comparable: 0.08 mm for the single-prism method and 0.12 mm for the multi-prism method. According to Section 2.4.2, the multi-prism method should offer slightly better result if the multi-prism platform was set fully vertically above the tube for every position. Since it was set on the tube in arbitrary position, $\sigma_{2}$ from Section 2.4.2 reduces the accuracy. On the other hand, the precision values using a single-prism were overestimated. The prism was in the same position with respect to the TS during the measurement; thus, the nominal centering accuracy affected the estimated line parameters instead of the residuals. 

The results from Figure 9 clearly show that using the multi-prism platform will allow us to determine the tube center with higher quality than using the single-prism method. Thus, this test confirms the theoretical assumptions about the stochastic models for both methods presented in Section 2.4.1 and Section 2.4.2 to some extent. The fact that identification error mostly affects only horizontal components is not exposed in stochastic model and pessimistic assumption about 1 mm error sphere input is also exceeded in laboratory test.

### 3.2. Case study and Field Measurements

The measurements took place at the Gorišek Brothers’ ski-flying hill in Planica (Slovenia). Besides the flying hill in Vikersund (Norway), Planica is the biggest ski-flying hill in the world, with a nominal hill size of HS=240 m. Due to the size of the object, the quality of hill preparation is extremely important for the safety of the athletes. The length of the inrun (Figure 1a) is 133.8 m and begins with a straight line (length: 8 m, inclination: $11.5^{{}^{\circ}}$) at the bottom, continues with first circle arc (length: 25 m, radius: 105.868 m) and the second circle arc (length: 34 m, radius: 178.188 m), and ends with a straight line (inclination: $35.1^{{}^{\circ}}$). The true geometry of the inrun must be determined with a precision of 5 mm for each coordinate component. Of course, the smaller the offsets, the fewer disturbances for the skiers. Two adjustment screws positioned at each meter of the tube allow for fine adjustments of the tube in the vertical and traverse directions (see Figure 1b).

A Leica Geosystems TS30 total station was used for the survey. The instrument’s nominal precision is $\sigma_{ISO\;17123-3}=0.5^{\prime \prime }$ for directions and $\sigma_{ISO\;17123-4}=0.6\;\mathrm{mm}\pm 1\;\mathrm{ppm}$ for distances. The measurements were carried out using the multi-prism and single-prism methods. Field work was done under favorable weather conditions in the autumn before the snow. Meteorological corrections were applied according to [25,26].

The primary z axis of the object coordinate system is defined by the zenith direction. Axes x and y are in the horizontal plane, with the x axis along the inrun of the Planica flying hill. 

**Figure 10:**
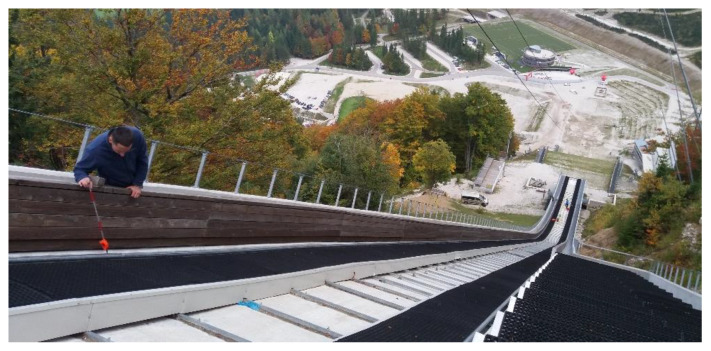
Measurements with the single-prism method on the inrun of Planica ski-flying hill.

First, the single-prism method was used. The points were signalized with a Leica mini GMP111-0 prism (Figure 3, Figure 10) using the shortest possible prism height of 10 cm. An ATR system for semi-automated measurements was used. On some parts of the tube, the top is designated by the groove. However, the longitudinal position above the adjustment screws must still be identified manually. To improve the accuracy of the measured points and eliminate potential instrumental errors, measurements were captured in both faces. In total, 136 points were measured on each tube. The main disadvantage of the single-prism method is its problematic definition of the characteristic point of the tube. It is also practically impossible is to repeat measurements on an identical point.

**Figure 11:**
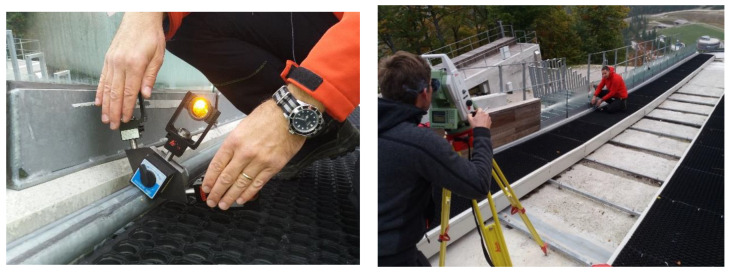
Measurements with the multi-prism method and the covering of two prisms during measurements on the inrun of Planica ski-flying hill.

Measurements were next made using the multi-prism platform (Section 2.1). The most important difference of the proposed platform is that the center of the pipe is determined instead of a point at the top of the tube, as it is done when using the single-prism method. The positions of all three prisms on the platform were measured from a single station for both faces along the tube. To ensure that the ATR system would not fix itself to an incorrect prism, two other prisms were covered (Figure 11).

### 3.3. Comparison of Both Measurement Methods

**Figure 12:**
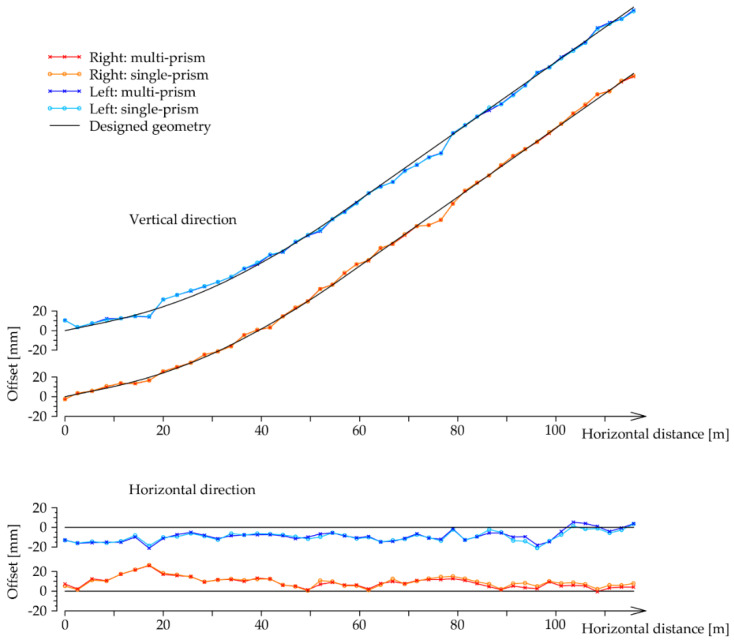
Offsets of the determined characteristic point C from its designed value in the horizontal and vertical direction for both methods (single- and multi-prism) and both inrun tubes (left and right).

**Figure 13:**
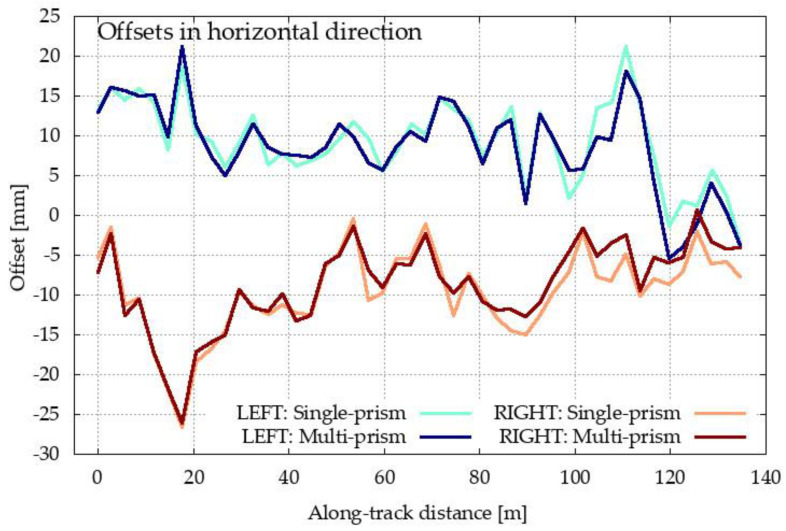
Offsets of the determined characteristic point C from its designed value in the horizontal direction for both methods (single- and multi-prism) and both inrun tubes (left and right).

**Figure 14:**
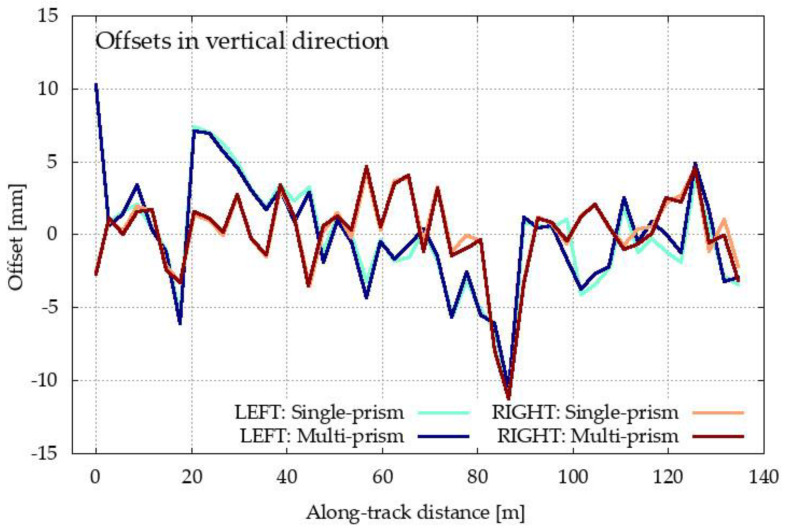
Offsets of the determined characteristic point C from its designed value in the vertical direction for both methods (single- and multi-prism) and both inrun tubes (left and right).

The measurements for both methods were processed and are represented bellow. In both cases, the characteristic point C was determined for each set up of the single- and multi-prism methods. The position of each calculated point C for both methods was compared to its true position (i.e., the designed position obtained from the construction project) [2]. Offsets of the calculated C positions with respect to their designed positions are depicted in Figure 12. Despite the very large scale for offsets, there are almost no differences in the graphs for both methods (single- and multi-prism) and both inrun tubes (left and right). We can see that the offsets are larger for the horizontal direction. Therefore, the results are shown in more detail in Figure 13 and Figure 14.

Figure 13 presents the offsets of point C in the horizontal direction for both methods and for both inrun tubes. The offset to the right of the inrun tube is defined by a positive sign, whereas the offset on the left of the inrun tube is defined with a negative sign. In the same way, in Figure 14, offsets of point C are presented in the vertical direction.

Figure 12, Figure 13 and Figure 14 present the offsets of the determined points C with respect to their designed values in the horizontal and vertical directions. The numerical values of the offsets may exceed several centimeters in the horizontal direction, thereby deforming the true geometry of the inrun by up to 5 cm. On the other hand, the offsets in the vertical directions are at most at a centimeter level. In both cases, the offsets are significantly larger than the differences between both methods. The statistical values of the differences between both methods for the left and right inrun tubes of Planica flying hill are presented in Table 2. 

In the horizontal direction, the differences are, on average, at the sub millimeter level, but with a standard deviation of a couple of millimeters. However, horizontal differences may span more than 5 mm, as seen from the values in Table 1 (maximum–minimum difference). In the vertical directions, the differences are smaller and are statistically identical on average, with the standard deviation bellow one millimeter and spanning a few millimeters.

According to the theoretical conclusions for both methods (Section 2) and the laboratory test (Section 3.1), the differences from Table 1 are mostly errors in the single-prism method. Differences are significantly higher in the horizontal direction, as shown in Figure 9 for the laboratory test. 

The quality of both methods is thoroughly presented in Section 2.4. The precision and unbiased determination of characteristic points is the main advantage of the proposed multi-prism method. The multi-prism method always assures that the determined center point is independent of the orientation/placement of the platform on the tube. On the other hand, the single-prism method presents two problems. The first relates to the repeatability of the selected point. It is practically impossible to place the prism on the same point several times. The second problem is determining the highest point on the tube. This process depends upon the surveyors’ subjective decisions. 

Since the random and instrumental errors apply equally to both methods, the differences between the results originate mostly from subjective characteristic point decisions for the single-prism method.

## 4. Discussion and Conclusions

Preparing the ski-flying hill is the most important step to prepare for World Cup ski-flying matches. Besides the safety of the competitors, this preparation must also ensure fair assessment conditions. There are strict rules governed by the international ski competition rules [1] and precise requirements regarding the shapes and sizes given by the Certificate of Flying hill [2]. This article studies the control measurements for the geometry of the upper part of the ski-flying hill, where ski-jumpers gain their speed and take off. It is important to ensure the adequate geometry of the inrun in situ. To achieve the best possible performance, an optimal control measurement methodology must be used.

The method currently being used is the classical polar method (single-prism), which poses several issues. Two of these issues are related to the measuring process, repeatability, and determination of the true point. Without physical stabilization of the geodetic point, it is impossible to repeat measurements several times on the same point. On the other hand, selection of the highest point on the tube of the flying hill inrun is dependent upon the subjective decision of the surveyor. The last issue is related to the methodology of point calculation. Since we are unable to perform redundant observations, a straightforward calculation of coordinates is the only possibility. No adjustments, statistical evaluations, or outlier detection are possible without using redundant observations and the least squares method. On the other hand, determination of the characteristic point/center of the tube is done by reducing the calculated prism height and radius of the tube. Based on the error propagation law, we have shown that the tube center is measured with approximately three times lower precision than the center of the prism. This error increases by moving from the prism to the tube center, mostly in the horizontal direction, while the vertical component is affected to a lesser extent.

In this article, we proposed a methodology based on a three-prism platform to eliminate the disadvantages of the single-prism method. The construction of the multi-prism platform provides us with:

- Unique placing of the platform on the tube;
- Accurate determination of the center point of the tube independent from the orientation of the platform on the tube.

Additionally, since three points were measured on the platform, we obtained redundant observations where the inner-geometry is known accurately and precisely. Thus, the possibility of using least squares adjustment, stochastic modelling, and outlier detection is assured.

We developed a processing methodology that is based on rigid body transformation and the least squares method. The solution to the stochastic model describes the statistical properties of the determined characteristic point of the tube. We have shown that, unlike a single-prism, the center point of a tube is obtained with higher precision than the measured centers of the prisms. This precision increases by 42% in the height direction and by 3% in the horizontal direction (the traverse to tube direction). For the multi-prism method, the precision of tube center in the traverse section of the tube is always determined with higher precision than the center of the prism.

This method was tested in a laboratory case study on a 1.4-m-long tube with known geometry. The results of the test show that the proposed multi-prism method is able to determine the characteristic point of the tube more precisely than the single-prism method, especially in the horizontal direction. Unlike the single-prism method, the multi-prism method is able to determine the center point of the tube with comparable precision in the both the vertical and horizontal directions. Similar results were obtained on the Planica flying hill for both tubes of the inrun. The results are comparable in the vertical direction but are significantly different in the horizontal direction.

The additional advantage of the multi-prism measurement method and calculation procedure is that the tube inclination is taken into account. If the inclination is known with a precision of at least 0.4°, then the positional precision of the characteristic point in all three coordinate axes can be better than 1.4 mm.

The proposed multi-prism method was able to eliminate most disadvantages of the single-prism method. The method resulted in higher precision and also higher accuracy due to the platform’s construction. The center point of the tube was determined independently of the platform’s orientation according to the tube; therefore, the present method is also unbiased. The comparative disadvantage of this method is its measuring time (three times longer), which should not pose a problem for tasks of such importance.

The proposed methodology for control measurements may also be interesting for other types of geometric monitoring where the geometric quality of round tubes needs to be controlled or evaluated.

## Figures and Tables

**Table 1:** Improvement of the along-tube positional precision of characteristic point C as a function of inclination precision.

Inclination Precision	$\mathrm{Factor}\;\mathrm{of}\;\sigma_{MP}$	$\sigma_{1}\;[\mathrm{mm}]$
no inclination	3.41	4.8
2°	2.63	3.7
1.5°	2.30	3.2
1.0°	1.81	2.5
0.5°	1.15	1.6
0.4°	1.01	1.4
0.3°	0.88	1.2

**Table 2:** Statistics on the differences between both methods (single- and multi-prism) for both inrun tubes (left and right).

	Horizontal Direction		Vertical Direction	
	**left tube**	**right tube**	**left tube**	**right tube**
average difference	0.6 mm	−0.9 mm	0.0 mm	−0.1 mm
difference standard deviation	1.9 mm	1.6 mm	0.7 mm	0.4 mm
min. value	−3.5 mm	−4.7 mm	−1.4 mm	−0.6 mm
max. value	5.7 mm	1.9 mm	2.7 mm	1.1 mm
